# Effects of Urea-Ammonium Nitrate Solution on Yield, N_2_O Emission, and Nitrogen Efficiency of Summer Maize Under Integration of Water and Fertilizer

**DOI:** 10.3389/fpls.2021.700331

**Published:** 2021-08-03

**Authors:** Baizhao Ren, Yanqing Guo, Peng Liu, Bin Zhao, Jiwang Zhang

**Affiliations:** State Key Laboratory of Crop Biology and College of Agronomy, Shandong Agricultural University, Tai'an, China

**Keywords:** *Zea mays* L, N fertilizer source, N loss, N_2_O warming potential, agronomic effectiveness

## Abstract

In order to clarify the effects of urea-ammonium nitrate solution (UAN) on the yield, nitrogen-use efficiency (NUE), and N_2_O emissions of summer maize under the condition of water and fertilizer integration, different types of nitrogen fertilizer were selected, namely, ordinary urea (urea) and UAN. Our results showed that the application of UAN was beneficial to improve the dry matter accumulation and the distribution of summer maize. Compared with urea treatment, the total nitrogen accumulation of UAN treatment was increased by 15.8%, and the harvest index was increased by 5.5%. The partial productivity, agronomic use efficiency, and recovery rate of nitrogen for UAN treatment were also increased by 9.1, 19.8, and 31.2%, respectively, compared to those of urea treatment. The soil nitrogen dependence rate treated with UAN was significantly decreased by 13.6%, compared to that of urea treatment. In addition, UAN was beneficial to reduce N_2_O emissions. The N_2_O warming potential (GWP_N2O_) and N_2_O greenhouse gas intensity (GHGI_N2O_) of urea treatment were 39.3 and 52.4% higher, compared to those of UAN treatment. The improvement of dry matter accumulation and distribution and nitrogen efficiency for UAN treatment were beneficial to increase the grain yield by 9.1%, compared to that of urea treatment. In conclusion, under the fertigation, the application of UAN favors higher yield and nitrogen uptake, with less soil nitrogen residue, higher NUE, and better environmental effect.

## Introduction

Reasonable nitrogen management plays an important role in ensuring good nitrogen supply during crop growth and development, coordinating the relationship between vegetative growth and reproductive growth, and achieving the high yield and quality of the crop (Stanger and Lauer, 2008; Fan et al., 2012). However, excessive nitrogen fertilization and the backward of fertilization technology used in current agriculture for pursuing high yield lead to a low utilization rate of nitrogen fertilizer. In addition, soil nitrogen is prone to volatilization, leaching, and denitrification, which will cause resource waste, and environmental pollution, thus adversely affecting agricultural sustainable development (Rowe et al., 2006; Zhang et al., 2008; Omonode et al., 2017). Nitrogen-use efficiency (NUE) in cereal grain production may be low owing to N losses induced by volatilization, denitrification, and leaching (Sylvester-Bradley and Kindred, 2009; Ercoli et al., 2013; Pampana and Mariotti, 2021). Traditional agricultural nitrogen fertilizer in China is dominated by urea, which has the problems such as excess capacity and low utilization rate (Ju and Gu, 2014), and the characteristics of instant dissolution and rapid dispersion (Zhang et al., 2013). It has been estimated that the direct loss of nitrogen fertilizer applied in traditional agriculture is 10–78%, and about 40% of nitrogen can be lost within a few days after application (Li et al., 2013). Therefore, how to improve the utilization rate of fertilizer, reduce the fertilizer rate, and develop new fertilizers with high efficiency and environmentally neutral has become a major issue in the modern agricultural science.

Urea-ammonium nitrate solution (UAN) integrates three nitrogen forms, namely, nitrate nitrogen, ammonium nitrogen, and amide nitrogen, and has been widely used in the European Union, the United States, Australia, and other countries (Millar et al., 2010). Inside, UAN currently accounts for 80% of the liquid fertilizers in the United States (Habibullah et al., 2017; Nikolajsen et al., 2020). UAN is the most efficient nitrogen source compared to urea, calcium ammonium nitrate (CAN), and anhydrous ammonium (AA), because it provides the maximum crop response and availability of soil inorganic nitrogen content (Gagnon and Ziad, 2010; Sundaram et al., 2019), while the soil nitrogen residual and surplus were less, compared to those of urea applications (Connella et al., 2011; Zhang et al., 2017; Wang et al., 2018). The application of UAN could significantly increase the grain yield of spring maize, promote the absorption and utilization of nitrogen, and reduce the residual amount of soil nitrogen (Wang et al., 2018).

At present, modern irrigation facilities are constantly updated and popularized. The agricultural area with water-saving facilities such as drip irrigation and sprinkler irrigation system is gradually increasing (Li, 2019). Water-fertilizer coupling technique, fertigation, is a relatively new agricultural method that allows applying water and fertilizer timely and appropriately, is conducive to nutrient absorption by plants, and gives full play to the effect of fertilizers. Fertigation can increase the grain yield by 20–50%, economize water by more than 40%, and improve the fertilizer utilization rate by more than 20%, compared to traditional fertilization techniques (Shen and Tian, 2013). UAN, as a liquid nitrogen fertilizer, is easy to mix with other nutrients or chemicals and is suitable for sprinkler fertigation technology. In addition, since there is no need for the granulation process of urea production, energy consumption can be significantly reduced. Halvorson and Del Grosso (2012) found that less N_2_O was released by applying UAN than urea in maize production, while some studies found no difference between UAN and urea treatments (Venterea et al., 2011; Sistani et al., 2014). However, the application of UAN in China is still in the initial stage. There are few studies on the effects of nitrogen fertilizer types on the grain yield, N losses, N_2_O warming potential, and agronomic effectiveness of summer maize under microspray fertigation. Our objective was to explore the effects of applying UAN or urea on the grain yield, NUE, and N_2_O emissions of summer maize under the fertigation, to highlight better N sources.

## Materials and Methods

### Plant Materials and Experimental Location

**Figure 1:**
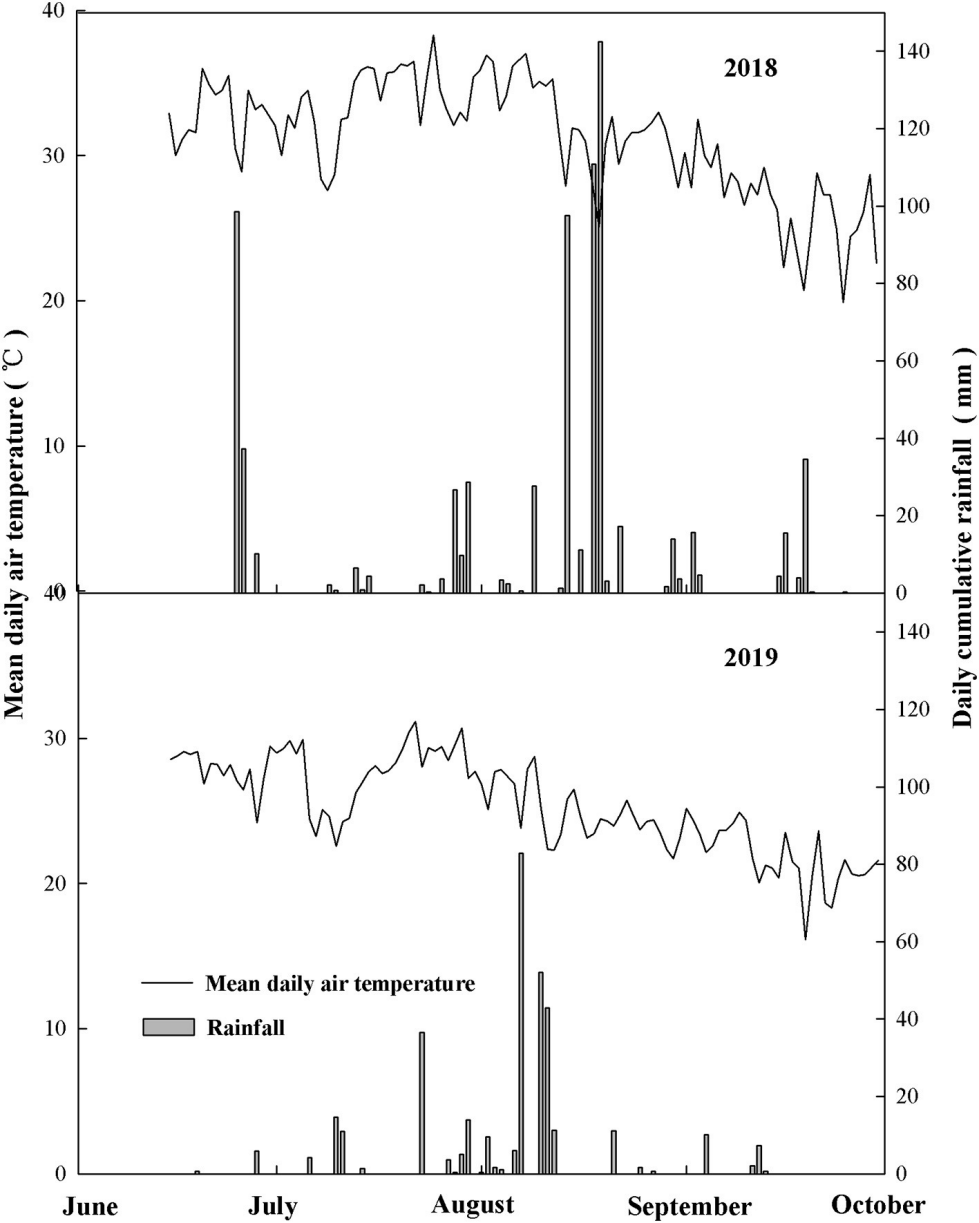
Weather conditions during maize season (from June to October) in 2018 and 2019.

This study was conducted in two maize seasons in 2018 and 2019 in the Key Laboratory of Crop Biology of Shandong Agricultural University and Mazhuang Town (35°99′ N, 117°01′ E) of Daiyue District, Taian City, Shandong Province. The topsoil (0–20 cm) type is brown loam with 12.6 g kg^−1^ of organic matter, 2.17 g kg^−1^ of total nitrogen, 5.86 g kg^−1^ of total phosphorus, 6.84 g kg^−1^ of total potassium, 24.57 mg kg^−1^ of nitrate nitrogen, and 3.81 mg kg^−1^ of ammonium nitrogen. The weather conditions of summer maize-growing season in the planting area are shown in Figure 1. The maize hybrid Denghai 618 (DH618) was selected as the experimental material. Maize was sown on June 15 and harvested on October 3, with a planting density of 67,500 plants hm^−2^.

### Experimental Design

Two fertilization types were set, namely, conventional solid nitrogen fertilizer–ordinary urea (46% nitrogen content) and water-soluble nitrogen fertilizer, UAN (32% nitrogen content, ratio of nitrate nitrogen, ammonium nitrogen, and amide nitrogen was 1:1:2, respectively). The unfertilized nitrogen treatment was used as control (N0). At the sixth leaf stage (V6) and 12th leaf stage (V12), the nitrogen fertilizer was sprayed by the micro-spraying method in a ratio of 4:6. Micro-spraying belts were laid between maize rows, and nitrogen fertilizer was injected into the pipeline with water (about 10 L m^2^) for spraying. N0 treatment was sprayed with the same amount of water. Phosphate fertilizer (P_2_O_5_) and potassium fertilizer (K_2_O) were applied one-off for all treatments to prepare soil before seeding. The rates of P_2_O_5_ and K_2_O were 52.5 and 67.5 kg ha^−1^, respectively. Each treatment was repeated three times, in a completely randomized design, and the plot area was 333.5 m^2^.

### Dry Matter Weight and NUE

At the physiological maturity stage (R6), five representative plant samples were randomly selected from each treated plot and divided into stem, leaf, and ear. Samples were placed in an oven at 105°C for degreening and then dried at 80°C to a constant weight. Nitrogen content of samples was measured with an AA3 continuous flow analyzer (SFA CFA FIA BRAN+LUEBBE III). NUE was calculated as follows (Zhao et al., 2010):

Nitrogen-use efficiency(NUE,%)=[100× (NF−NC)]/NA

where NF is the nitrogen content (kg) extracted from the plants obtained from the fertilization plot. NC is the nitrogen content (kg) extracted from the plants of the control plot. NA is the amount of nitrogen applied in different plots (kg).

Nitrogen partial factor productivity from (NPFP, kg kg^−1^) = grain yield/N rate

Nitrogen agronomic efficiency (NAE, kg kg^−1^) = (grain yield with applied N–grain yield with applied N0)/N rate

Nitrogen recovery efficiency (NRE, kg kg^−1^) = (total N uptake by plant with applied N–total N uptake by plant with applied N0)/N application amount

Soil nitrogen dependency rate (SNDR, %) = (total N uptake by plant with applied N0/total N uptake by plant with applied N × 100)

Nitrogen harvest index (NHI, %) = grain N amount/total N amount of aboveground organ × 100

Harvest index (HI, %) = grain dry weight/total dry weight of aboveground organ.

### Soil N_2_O Fluxes Measurements

N_2_O fluxes were measured by the closed-chamber gas chromatography (Zhang et al., 2010). Three chambers were set between maize rows for each treatment. The closed chamber was enclosed with plastic sheets and had dimensions of 0.35m × 0.35m × 0.2m (length × width × height). The exterior of chamber was insulated using sponge material and aluminum foil, and an air vent was installed in the middle of the chamber. A pedestal was placed under the chamber, and the base was sealed using water to ensure that the external environment did not affect the interior of chamber when gases were extracted. Gas samples (50 mL) were collected using glass syringes from the chamber headspace at 0, 10, 20, and 30 min after the soil sample was covered. Concentrations of N_2_O in the gas samples were detected using an Agilent GC7890 gas chromatograph (Agilent, Santa Clara, CA, United States) with an electron capture detector (ECD). Gas samples were collected every other week in 2018. In 2019, they were collected once every 2 days within a week after fertilization and then once a week for collection (Zhang et al., 2010).

For each gas, the flux was calculated as follows:

$$\begin{array}{l} J=dc/dt\times \;\left(M/V_{0}P\right)/\left(P_{0}T_{0}/TH\right), \end{array}$$

where *J* is the flux (mg m^−2^ h^−1^), *dc/dt* is the change in gas concentration (*c*, mg m^−3^) against time (*t*, hour), *M* is the molar mass (g mol^−1^) of each gas, *P* is the atmospheric pressure (kPa), *T* is the absolute temperature (°*K*) during sampling, *H* is the height (m) of headspace in the chamber, and *V*_0_, *T*_0_, and *P*_0_ are the gas molar volume (m^3^ mol^−1^), absolute air temperature (°*K*), and atmospheric pressure (kPa), respectively, under standard conditions.

### N_2_O Emission Coefficient, Global Warming Potential, and Greenhouse Gas Emission Intensity

N_2_O coefficient was used to evaluate the percentage of N_2_O emission in fertilizers (Mazzetto et al., 2020):

$$\begin{array}{l} {\text{N}}_{\text{ef}}=\left(\text{Nf}-\text{Nc}\right)/\text{NA}\times 100 \end{array}$$

where N_*ef*_ was the N_2_O emission factor (%) in the fertilizer, NF was the N_2_O emission in the nitrogen-applied plot (kg hm^−2^), NC was the N_2_O emission in the nonfertilized plot (kg hm^−2^), and NA was the nitrogen application amount (kg) in different plots.

N_2_O warming potential (GWP) represents the potential effect of N_2_O on global warming, which at the 100-year warming scale was 265 times that of CO_2_ (Kumar et al., 2007). The calculation formula of GWP was as follows:

$$\begin{array}{l} \text{GW}{\text{P}}_{\text{N}2\text{O}}={\text{f}}_{\text{N}2\text{O}}\times 265 \end{array}$$

where GWP_N2O_ was the warming potential of N_2_O (kg hm^−2^), and f_NO2_ was the net emission of N_2_O (kg hm^−2^).

N_2_O greenhouse gas intensity (GHGI_N2O_) was an evaluation index of low carbon agriculture at the present stage, considering both crop yield and comprehensive net greenhouse effect. The GHGI_N2O_ was calculated as follows (Zhang et al., 2015):

$$\begin{array}{l} \text{GG}{\text{I}}_{\text{N}2\text{O}}=\text{GW}{\text{P}}_{\text{N}2\text{O}}/\text{Y} \end{array}$$

where Y represents the crop yield (kg hm^−2^).

### Soil ${\text{NH}}_{4}^{+}$-N and ${\text{NO}}_{3}^{\text{–}}$-N

Soil samples were divided into three layers from 0 to 60 cm, each one with a height of 20 cm. Each soil sample (60 cm length by 20 cm depth) was extracted by using an earth drill. Soil sample of each layer was placed by an earth drill into a Ziploc bag at V6, V12, VT, VT+30 d, and R6 stages. Soil ${\text{NH}}_{4}^{+}$-N and ${\text{NO}}_{3}^{-}$-N were extracted with 1 M KCl and filtered through a 0.45-μm membrane to remove insoluble particles. The contents of soil ${\text{NH}}_{4}^{+}$-N and ${\text{NO}}_{3}^{-}$-N were measured with an AA3 Continuous Flow Analytical System (Zhu et al., 2015). Three replicate soil samples were collected in each plot.

### Crop Yield and Production Value

To determine the maize yield and ear traits, 30 ears were harvested at the physiological maturity stage (R6) from three rows at the center of each plot. All kernels were air-dried, and the grain yield was measured at 14% moisture, the standard moisture content of maize in storage or for sale in China (GB/T 29890-2013).

According to the local market price (urea, $ 0.48 kg^−1^ N; phosphorus fertilizer, $ 0.82 kg^−1^ P_2_O_5_; potassium fertilizer, $ 0.63 kg^−1^ K_2_O; UAN, 0.73 kg^−1^ N; corn, $ 0.25 kg^−1^), the output value, economic benefits, and the ratio of production to investment were calculated. In this paper, only fertilizer input costs were calculated. Other inputs (including seeds, pesticides, machinery, labor, etc.) were the same and not included (Zhang et al., 2018).

### Statistical Analysis

Data were treated by ANOVA. The main effects of year, fertilization, and their interactions were tested for the grain yield, dry matter weight, NUE, and N_2_O emission using SPSS17.0 (SPSS Institute Inc. United States). Significantly different means were separated at the 0.05 probability level by the least significant difference test.

## Results

### Grain Yield

The application of UAN increased the grain yield of summer maize under fertigation. There were no significant year × fertilization interaction effects on the grain yield (Table 1). In both years, the grain yield of UAN treatment was increased by 9.1%, compared to urea treatment. The increase in maize yield was mainly due to the significant increase in grain number per ear under UAN treatment, which was 5.1% higher than that of urea treatment across years. In addition, the 1,000-grain weight of UAN treatment increased, while there was no significant difference between UAN and urea treatments (Table 1). Moreover, the application of UAN improved the production value of summer maize under microspray fertigation. The output value and economic benefits of UAN treatment improved by 9.1 and 8.2% across years, respectively, compared to those of urea treatment. However, there were no significant differences in the ratio of production to investment among treatments (Table 1).

**Table 1:** Effects of nitrogen fertilizer types on grain yield and production values of summer maize.

**Year**	**Treatment**	**Ears (No·ha^**−1**^)**	**Kernels number per ear**	**1000 grain weight** **(g)**	**Grain yield (kg·ha^**−1**^)**	**Production value** **($ ha^**−1**^)**	**Economic benefits** **($ ha^**−1**^)**	**The ratio of production to investment**
2019	N0	69,997	319c	278b	6,197c	1,549c	1,378c	9.1b
	Urea	72,667	547b	339a	13,479b	3,370b	3,098b	12.4a
	UAN	74,331	582a	345a	14,938a	3,735a	3,410a	11.5a
2018	N0	69,046	383c	318b	8,396c	2,099c	1,928c	12.3a
	Urea	70,500	526b	364a	13,478b	3,370b	3,098b	12.4a
	UAN	71,445	546a	371a	14,469a	3,617a	3,293a	11.2a
**ANOVA**								
Year (Y)		NS	^*^	NS	NS	NS	NS	NS
Treatment (T)		^*^	^*^	^*^	^*^	^*^	^*^	NS
Y×T		NS	NS	^*^	NS	NS	NS	NS

*Values followed by a different small letter within a column are significantly different at 5% probability level. Differences between treatments were calculated within the hybrids for each particular year. NS, Not significant*.

* *Significant at the 0.05 probability level*.

### Dry Matter Accumulation and Distribution

Urea-ammonium nitrate solution application was beneficial to the dry matter accumulation and the distribution of summer maize under fertigation. The total dry weight of UAN treatment at R6 stage increased by 11.2% across years, compared to that of urea treatment. Likely, the dry matter weight of each organ for UAN treatment significantly increased compared with that of urea treatment. The dry matter weight of stem, leaf, and ear under UAN treatment also increased by 10.6, 8.6, and 12.0% across years, respectively, compared to those of urea treatment. In addition, the application of UAN increased the harvest index by 2.8%, compared to that of urea treatment (Table 2).

**Table 2:** Effects of nitrogen fertilizer types on dry matter accumulation and distribution of summer maize.

**Year**	**Treatment**	**Total dry matter**	**Stem**		**Leaf**		**Ear**		**Harvest Index**
		**(g·plant^**−1**^)**	**(g·plant^**−1**^)**	**(%)**	**(g·plant^**−1**^)**	**(%)**	**(g·plant^**−1**^)**	**(%)**	**(HI)**
2019	N0	187.9c	48.5c	25.8	23.8c	12.7	115.6c	61.5	0.54b
	Urea	324.3b	77.6b	23.9	38.7b	11.9	208.0b	64.1	0.57a
	UAN	360.a1	85.0a	23.6	44.6a	12.4	230.6a	64.0	0.58a
2018	N0	253.7c	86.3c	34.0	32.1b	12.7	135.3c	53.3	0.46c
	Urea	334.3b	98.4b	29.4	39.2a	11.7	196.7b	58.8	0.53b
	UAN	372.5a	109.9a	29.5	40.0a	10.7	222.6a	59.8	0.55a
**ANOVA**									
Year (Y)		^*^	^*^		NS		NS		NS
Treatment (T)		^*^	^*^		^*^		^*^		^*^
Y×T		^*^	NS		NS		NS		^*^

*Values followed by a different small letter within a column are significantly different at 5% probability level. Differences between treatments were calculated within the hybrids for each particular year. NS, Not significant*.

* *Significant at the 0.05 probability level*.

### Nitrogen Accumulation and Distribution

Urea-ammonium nitrate solution application increased N accumulation and the distribution of summer maize under fertigation. The total N accumulation of UAN treatment at the physiological maturity stage (R6) increased by 15.8%, compared to that of urea treatment. The N accumulation of stem, leaf, and ear for UAN treatment was, respectively, 27.2, 16.9, and 20.4% higher than those of urea treatment. In addition, the application of UAN increased N harvest index by 5.5% across years, compared to that of urea treatment (Table 3).

**Table 3:** Effects of nitrogen fertilizer types on nitrogen accumulation and distribution of summer maize.

**Year**	**Treatment**	**Total N**	**Leaf N**	**Stem N**	**Ear N**	**N harvest index**			
		**(g·plant^**−1**^)**	**(g·plant^**−1**^)**	**(%)**	**(g·plant^**−1**^)**	**(%)**	**(g·plant^**−1**^)**	**(%)**	**(NHI)**
2019	N0	1.56c	0.30c	19.27	0.20c	12.87	1.01c	64.65	0.61b
	Urea	3.48b	0.62b	17.79	0.44b	12.60	2.24b	64.44	0.61b
	UAN	4.02a	0.73a	18.08	0.58a	14.31	2.72a	67.62	0.65a
2018	N0	1.40c	0.25c	17.91	0.19c	13.63	0.91c	64.90	0.61c
	Urea	3.07b	0.54b	17.55	0.42b	13.62	2.02b	65.89	0.63b
	UAN	3.56a	0.63a	17.60	0.52a	14.47	2.42a	67.93	0.65a
**ANOVA**									
Year (Y)		NS	^*^		NS		NS		NS
Treatment (T)		^*^	^*^		^*^		^*^		^*^
Y×T		^*^	NS		NS		^*^		NS

*Values followed by a different small letter within a column are significantly different at 5% probability level. Differences between treatments were calculated within the hybrids for each particular year. NS, Not significant*.

* *Significant at the 0.05 probability level*.

### N Efficiency

Urea-ammonium nitrate solution application was beneficial to the improvement of N efficiency of summer maize under fertigation. N partial factor productivity, N agronomic use efficiency, and N recovery efficiency of UAN treatment increased by 9.1, 19.8, and 31.2% across years, respectively, compared to those of urea treatment. In addition, the soil N dependence rate of UAN treatment significantly decreased by 13.6% across years, compared to that of urea treatment (Table 4).

**Table 4:** Effects of nitrogen fertilizer types on nitrogen efficiency of summer maize.

**Year**	**Treatment**	**NPFP (kg/kg)**	**NAE (kg/kg)**	**SNDR (%)**	**NRE (%)**
2019	Urea	64.19b	34.68b	44.87a	66.33b
	UAN	71.13a	41.62a	38.82b	87.04a
2018	Urea	64.18b	24.20b	45.64a	55.99b
	UAN	68.90a	28.92a	39.34b	73.45a
**ANOVA**					
Year (Y)		NS	^*^	NS	^*^
Treatment (T)		^*^	^*^	^*^	^*^
Y×T		NS	^*^	NS	NS

*NPFP, Nitrogen partial factor productivity; NAE, Nitrogen agronomic efficiency; SNDR, Soil nitrogen dependency rate; NRE, Nitrogen recovery efficiency*.

*Values followed by a different small letter within a column are significantly different at 5% probability level. Differences between treatments were calculated within the hybrids for each particular year. NS, Not significant*.

* *Significant at the 0.05 probability level*.

### N_2_O Emission and Warming Potential

**Figure 2:**
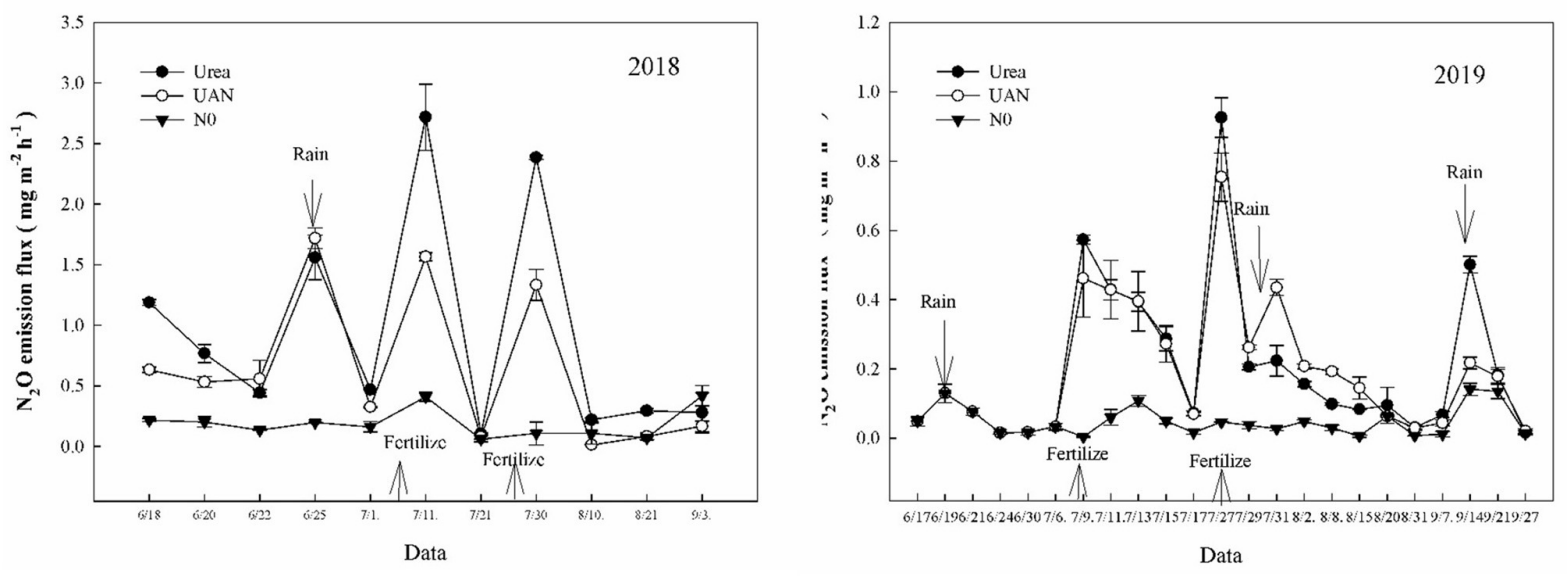
Effects of nitrogen fertilizer types on soil N_2_O emission.

The application of N fertilizer increased the N_2_O emission fluxes. The N_2_O emission peak of each treatment appeared after applying nitrogen, while the emission rate of UAN treatment was significantly lower than that of urea treatment under fertigation (Figure 2). As can be seen from Table 5, the cumulative emission flux of N_2_O for urea treatment was significantly higher than that of UAN treatment. The cumulative emission flux of N_2_O for urea treatment was increased by 39.3% on average, compared to that of UAN treatment. Moreover, the N_2_O emission coefficient (N_*ef*_) of urea treatment was significantly higher than that of UAN. The increase of N_2_O emission flux resulted in a significant increase in GWP and GHGI by 39.3 and 52.4% for urea treatment across years, respectively, compared to those of UAN treatment (Table 5).

**Table 5:** Effects of nitrogen fertilizer types on N_2_O emission, N_2_O emission coefficient, GWP, and GHGI.

**Years**	**Treatments**	**Cumulative N**_**2**_**O emission (kg N**_**2**_O·ha^**−1**^**)**	**N**_***ef***_ **(%)**	**GWP**_**N2O**_ **(CO**_**2-eq**_ **·** **ha** ^**−1**^ **)**	**GHGI**_**N2O**_ **(kg kg** ^**−1**^ **)**
2018	N0	3.4c	–	901.0c	0.15c
	Urea	21.4a	8.57a	5,671.0a	0.42a
	UAN	13.0b	4.57b	3,445.0b	0.23b
2019	N0	1.2c	–	318.0c	0.04c
	Urea	4.9a	1.76a	1,298.5a	0.10a
	UAN	4.3b	1.48b	1,139.5b	0.08b
**ANOVA**					
Year (Y)		^*^	^*^	^*^	^*^
Treatment (T)		^*^	^*^	^*^	^*^
Y×T		^*^	NS	^*^	NS

*Values followed by a different small letter within a column are significantly different at 5% probability level. Differences between treatments were calculated within the hybrids for each particular year. NS, Not significant*.

* *Significant at the 0.05 probability level*.

### Soil ${\text{NO}}_{3}^{\text{–}}$-N and ${\text{NH}}_{4}^{+}$-N Contents

**Figure 3:**
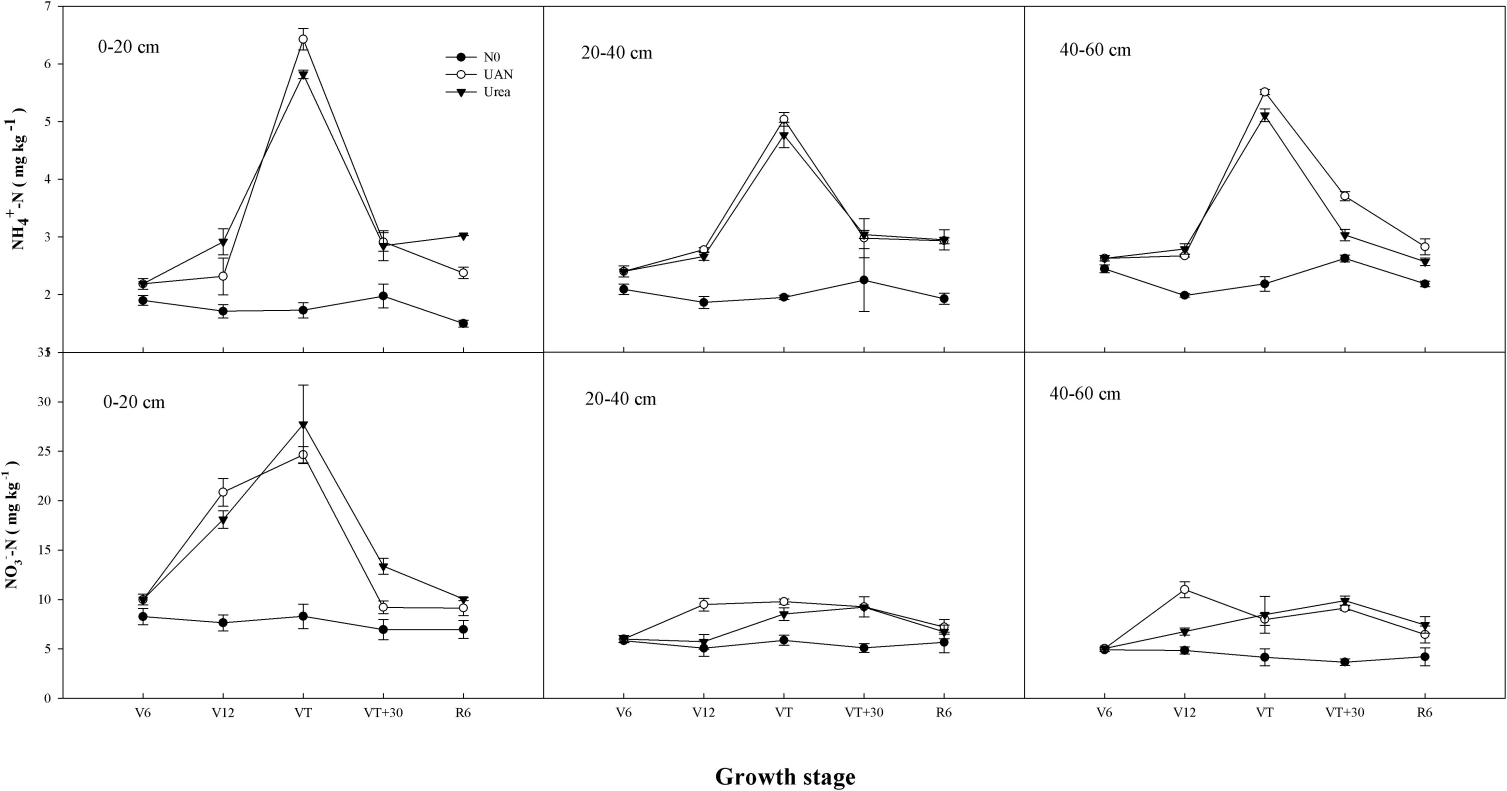
Effects of nitrogen fertilizer types on soil ${\text{NO}}_{3}^{-}$-N and ${\text{NH}}_{4}^{+}$-N content.

The contents of ${\text{NO}}_{3}^{-}$-N and ${\text{NH}}_{4}^{+}$-N of control plots (N0) remained at a low level and fluctuated little. After fertilization, the contents of ${\text{NO}}_{3}^{-}$-N and ${\text{NH}}_{4}^{+}$-N in soil increased and decreased with the deepening of soil layer. Compared with urea treatment, the contents of ${\text{NO}}_{3}^{-}$-N and ${\text{NH}}_{4}^{+}$-N for UAN treatment in 0–20 cm soil layer were significantly lower by 9.0 and 7.3%, respectively However, with the deepening of soil layer, the contents of ${\text{NO}}_{3}^{-}$-N and ${\text{NH}}_{4}^{+}$-N of UAN treatment increased significantly, compared to that of urea treatment. In the 20- to 40-cm soil layer, the contents of ${\text{NO}}_{3}^{-}$-N and ${\text{NH}}_{4}^{+}$-N in UAN treatment were, respectively, 22.1 and 2.6% higher than those in urea treatment. In the 40- to 60-cm soil layer, the contents of ${\text{NO}}_{3}^{-}$-N and ${\text{NH}}_{4}^{+}$-N for UAN treatment increased by 9.2 and 13.3%, compared to those of urea treatment, respectively (Figure 3).

## Discussion

Nitrogen (N)is one of the nutrient elements with the greatest demand for maize. N fertilizer not only has significant effects on the maize growth and yield formation, but also affects the environmental quality (Meng et al., 2012). Indeed, excessive application of N fertilizer not only caused a decrease in NUE of maize, but also increased the risk of N losses. Therefore, the rational application of N fertilizer is particularly important in maize production (Fan et al., 2012; Meng et al., 2012). Previous studies have shown that the rational use of UAN under different fertilization methods can reduce N loss and ammonia volatilization and increase the utilization efficiency of N fertilizer (Kelley and Sweeney, 2005; Abalos et al., 2016; Ransom et al., 2016). Our results showed that N accumulation and grain N distribution ratio of summer maize for UAN treatment were significantly higher than those of urea treatment. It indicates that UAN was beneficial to the redistribution of N from vegetative organs to reproductive organs, thus improving NUE. Moreover, N uptake efficiency, N agronomic use efficiency, and N partial productivity of UAN treatment were significantly higher than those of urea treatment, showing that UAN could effectively improve N efficiency, coordinate the supply balance of N nutrient, and increase the crop yield of summer maize. All these contribute to reduce the ineffective loss of N, and consequently in the reduction of environment pollution, under fertigation. N harvest index reflected the distribution of N in grain and vegetative organs at R6 stage. Under fertigation, UAN treatment increases N recovery rate and decreases soil N-dependent rate, promoting the uptake and use of N fertilizer, and grain N. As a result, N harvest and NUE were effectively improved and then significantly increased the grain yield of summer maize for UAN treatment, which was eventually beneficial to the increase in output value. Although the market price of UAN was higher than that of urea (Table 1), higher economic benefits can be obtained for the reason that the increasing range of the output value for UAN treatment was much greater than the increasing range of the input value.

N metabolism is a basic plant physiological process. The content and proportion of different forms of N among organs can reflect the nutrient status and physiological function of crop N (Baligar et al., 2001). Our results showed that the application of UAN significantly increased the N accumulation in leaves, stems, grains, and other organs under the condition of fertigation, which could improve leaf N assimilation, and crop physiological function, thus increasing the grain yield. The combined application of ${\text{NH}}_{4}^{+}$-N and ${\text{NO}}_{3}^{-}$-N can improve the photosynthetic efficiency of plants, thus achieving higher biomass and yield. First, the combination of ${\text{NH}}_{4}^{+}$-N and ${\text{NO}}_{3}^{-}$-N can make a rational and efficient use of the accumulated carbohydrates in each growth part of the plant and enable plants to store more nitrogen with less energy consumption. Second, the combined application of ${\text{NH}}_{4}^{+}$-N and ${\text{NO}}_{3}^{-}$-N could regulate the pH value of the rhizosphere, which was conducive to maintain the availability of phosphorus and trace elements, and protect soil ecological environment (Hinsinger et al., 2003; Hawkesford et al., 2011). The main difference between UAN and urea was the N form. UAN contains three forms of N, namely, ${\text{NH}}_{4}^{+}$-N, ${\text{NO}}_{3}^{-}$N, and amide nitrogen. However, urea only contains amide nitrogen, and most of amide nitrogen should hydrolyze into ${\text{NH}}_{4}^{+}$-N under the action of urease. At the same time, ${\text{NH}}_{4}^{+}$-N would undergo nitrification and oxidize to ${\text{NO}}_{3}^{-}$-N for plant uptake and utilization under aerobic conditions. Our results showed that the application of UAN was conducive to N transport and distribution, thus helping to improve the photosynthetic physiological characteristics associated with N, and reasonably and effectively use carbohydrates. As a result, dry matter accumulation increased significantly, while the allocation proportion of grain dry matter was improved, eventually leading to the increase in maize yield. In addition, UAN could give a full play to the respective advantages of different forms of nitrogen, which might also be the main reason for the increase in maize yield and NUE under fertigation conditions.

Soil ${\text{NO}}_{3}^{-}$-N and ${\text{NH}}_{4}^{+}$-N were the substrates for nitrification and denitrification, respectively. In a certain concentration range, the content of ${\text{NO}}_{3}^{-}$-N was positively correlated with the denitrification rate. After fertilization, the contents of soil ${\text{NO}}_{3}^{-}$-N and ${\text{NH}}_{4}^{+}$-N increased significantly, providing sufficient nitrogen sources for nitrification and denitrification, and ultimately promoted the increase in N_2_O emissions (Rizhiya et al., 2007; Gao et al., 2019). The cumulative emission of N_2_O for urea treatment was significantly higher than that of UAN treatment, which might be related to the different forms and conversion pathways of nitrogen. N_2_O is an important greenhouse gas, of which global warming potential is 300 times that of CO_2_ (IPCC, 2013). Farmland ecosystem is the main source of atmospheric N_2_O, contributing 6.2 Tg N_2_O–N·a^−1^ to global N_2_O emissions (17.7 Tg N_2_O–N·a^−1^), accounting for about one-third of global N_2_O emissions (Kroeze et al., 1999). Microbial nitrification and denitrification are the main N_2_O generation pathways in soil, which are affected by soil moisture content, temperature, aeration, ammonium and nitrate nitrogen concentrations, mineralizable carbon content, and pH (Sahrawat and Keeney, 1986; Granli and Bockman, 1994). Our results showed that the application of UAN significantly reduced N_2_O emission and significantly decreased the amount and intensity of net greenhouse gases emissions, under fertigation. The N_2_O emission coefficient (N_*ef*_) of urea treatment was significantly higher than that of UAN treatment. The increase of N_2_O emission flux resulted in the significant increase of GWP and GHGI, thus aggravating environmental pollution. In conclusion, UAN application, under fertigation, was beneficial to reduce soil greenhouse gas emissions, thus effectively alleviating the impact of nitrogen fertilizer on agricultural greenhouse effect.

## Conclusion

Under the integrated condition of water and fertilizer, the application of UAN was conducive to increase nitrogen accumulation, improve the utilization rate of fertilizer, and reduce soil N_2_O emission appropriately, thus improving the grain yield and environmental and economic benefits.

## Data Availability Statement

The original contributions presented in the study are included in the article/supplementary material, further inquiries can be directed to the corresponding author/s.

## Author Contributions

JZ and BR initiated and designed the research. YG performed the experiments. BR analyzed the data and wrote the manuscript. JZ, PL, and BZ revised and edited the manuscript and also provided advice on the experiments. All authors contributed to the article and approved the submitted version.

## Conflict of Interest

The authors declare that the research was conducted in the absence of any commercial or financial relationships that could be construed as a potential conflict of interest.

## Publisher's Note

All claims expressed in this article are solely those of the authors and do not necessarily represent those of their affiliated organizations, or those of the publisher, the editors and the reviewers. Any product that may be evaluated in this article, or claim that may be made by its manufacturer, is not guaranteed or endorsed by the publisher.

## References

[B1] Abalos D. Jeffery S. Druy C. F. Wagner-Riddlea C. (2016). Improving fertilizer management in the U.S. and Canada for N_2_O mitigation: understanding potential positive and negative side-effects on corn yield. Agric. Ecosyst. Environ. 221, 214–221. 10.1016/j.agee.2016.01.044

[B2] Baligar V. C. Fageria N. K. He L. (2001). Nutrient use efficiency in plants. Commun. Soil Sci. Plant Anal. 32, 921–950. 10.1081/CSS-100104098

[B3] Connella J. A. Hancock D. W. Durhama R. G. Cabrera M. L. Harris G. H. (2011). Comparison of enhanced-efficiency nitrogen fertilizers for reducing ammonia loss and improving bermudagrass forage production. Crop Sci. 51, 2237–2248. 10.2135/cropsci2011.01.0052

[B4] Ercoli L. Ercoli L. Masoni A. Pampana S. Mariotti M. Arduini I. (2013). As durum wheat productivity is affected by nitrogen fertilization management in Central Italy. Eur. J. Agron. 44, 38–45. 10.1016/j.eja.2012.08.005

[B5] Fan M. S. Shen J. B. Yuan L. Jiang R. F. Chen X. P. Davies W. J.. (2012). Improving crop productivity and resource use efficiency to ensure food security and environmental quality in China. J. Exp. Bot. 63, 13–24. 10.1093/jxb/err248 21963614

[B6] Gagnon B. Ziad N. (2010). Grain corn and soil nitrogen responses to sidedress nitrogen sources and application. Agron. J. 103, 1014–1022. 10.2134/agronj2010.0011

[B7] Gao F. Li B. Ren B. Z. Zhao B. Liu P. Zhang J. W. (2019). Effects of residue management strategies on greenhouse gases and yield under double cropping of winter wheat and summer maize. Sci. Total Environ. 687, 1138–1146. 10.1016/j.scitotenv.2019.06.146 31412450

[B8] Granli T. Bockman O. C. (1994). Nitrous oxide from agriculture. Norwegian J. Agric. Sci. 12, 1–128.

[B9] Habibullah H. Nelson K. A. Motavalli P. P. (2017). Assessing management of nitrapyrin with urea ammonium nitrate fertilizer on corn yield and soil nitrogen in a poorly-drained claypan soil. J. Agric. Sci. 9, 17–29. 10.5539/jas.v9n11p17

[B10] Halvorson A. D. Del Grosso S. J. (2012). Nitrogen source and placement effects on soil nitrous oxide emissions from no-till corn. J. Environ. Qual. 41, 1349–1360. 10.2134/jeq2012.0129 23099926

[B11] Hawkesford M. Horst W. Kichry T. Lambers H. Schjoerring J. Møller I. S.. (2011). Functions of macronutrient, in Marschners Mineral Nutrition of Higher Plants, ed. Marschner P. (Waltham: Academic Press), 135–189.

[B12] Hinsinger P. Plassard C. Tang C. Jaillard B. (2003). Origins of root-mediated pH changes in the rhizosphere and their responses to environmental constrains: a review. Plant Soil 248, 43–59. 10.1007/978-94-010-0243-1_4

[B13] IPCC (2013). Climate Change 2013: The Physical Science Basis. Working Group I Contribution to the IPCC 5th Assessment Report. IPCC, Cambridge; New York, NY.

[B14] Ju X. T. Gu B. J. (2014). Status-quo, problem and trend of nitrogen fertilization in China. J. Plant Nutr. Fertilizer 20, 783–795. 10.11674/zwyf.2014.0401

[B15] Kelley K. W. Sweeney D. W. (2005). Tillage and urea ammonium nitrate fertilizer rate and placement affects winter wheat following grain sorghum and soybean. Agron. J. 97, 690–697. 10.2134/agronj2004.0156

[B16] Kroeze C. Mosier A. Bouwman L. (1999). Closing the global N_2_O budget: a retrospective analysis 1500-1994. Global Biogeochem. Cycles 13, 1–8. 10.1029/1998GB900020

[B17] Kumar P. Martino D. Smith P. Al E. (2007). Chapter 8: Agriculture, in IPCC, 2007: Climate Change 2007: Mitigation of Climate Change. Contribution of Working Group III to the Fourth Assessment Report of the Intergovernmental Panel on Climate Change (New York, NY: Cambridge).

[B18] Li D. Watson C. J. Yan M. J. Lalor S. Rafique R. Hyde B.. (2013). A review of nitrous oxide mitigation by farm nitrogen management in temperate grassland-based agriculture. J. Environ. Manage. 128, 893–903. 10.1016/j.jenvman.2013.06.026 23880433

[B19] Li F. Y. (2019). Research progress on urea ammonium nitrate solution. Chem. Enterprise Manage. 22, 54–55. 10.3969/j.issn.1008-4800.2019.22.035

[B20] Mazzetto A. M. Styles D. Gibbons J. Arndt C. Misselbrook T. Chadwick D. (2020). Region-specific emission factors for Brazil increase the estimate of nitrous oxide emissions from nitrogen fertiliser application by 21%. Atmos. Environ. 230:117506. 10.1016/j.atmosenv.2020.117506

[B21] Meng Q. F. Chen X. P. Zhang F. S. Cao M. H. Cui Z.-L. Bai J.-S.. (2012). In-season root-zone nitrogen management strategies for improving nitrogen use efficiency in high-yielding maize production in China. Pedosphere 22, 294–303. 10.1016/S1002-0160(12)60016-2

[B22] Millar N. Robertson G. P. Grace P. R. Gehl R. J. Hoben J. P. (2010). Nitrogen fertilizer management for nitrous oxide (N_2_O) mitigation in intensive corn (Maize) production: an emissions reduction protocol for US Midwest agriculture. Mitig. Adapt. Strateg. Glob. Change 15, 185–204. 10.1007/s11027-010-9212-7

[B23] Nikolajsen M. T. Pacholski A. S. Sommer S. G. (2020). Urea ammonium nitrate solution treated with inhibitor technology: effects on ammonia emission reduction, Wheat Yield, and Inorganic N in Soil. Agronomy 10:161. 10.3390/agronomy10020161

[B24] Omonode R. A. Halvorson A. D. Bernard G. Vyn T. J. (2017). Achieving lower nitrogen balance and higher nitrogen recovery efficiency reduces nitrous oxide emissions in North America's maize cropping systems. Front. Plant Sci. 8:1080. 10.3389/fpls.2017.01080 28690623 PMC5481850

[B25] Pampana S. Mariotti M. (2021). Durum wheat yield and N uptake as affected by N source, timing, and rate in two mediterranean environments. Agronomy 11:1299. 10.3390/agronomy11071299

[B26] Ransom J. Simsel S. Schatz B. Eriksmoen E. Mehring G. Mutukwa I. (2016). Effects of a post-anthesis foliar application of nitrogen on grain protein and milling and baking quality of spring wheat. Am. J. Plant Sci. 7, 2505–2514. 10.4236/ajps.2016.717218

[B27] Rizhiya E. Bertora C. Vliet P. C. Kuikman P. J. Faber J. H. Groenigen J. W. (2007). Earthworm activity as a determinant for N_2_O emission from crop residue. Soil Biol. Biochem. 39, 2058–2069. 10.1016/j.soilbio.2007.03.008

[B28] Rowe E. C. Evans C. D. Emmett B. A. Reynolds B. Helliwell R. C. Coull M. C.. (2006). Vegetation type affects the relationship between soil carbon to nitrogen ratio and nitrogen leaching. Water Air Soil Pollut. 177, 335–347. 10.1007/s11270-006-9177-z

[B29] Sahrawat K. L. Keeney D. R. (1986). Nitrous oxide emission from soils. Adv. Soil Sci. 4, 103–148. 10.1007/978-1-4613-8612-4_2

[B30] Shen H. Tian J. C. (2013). Optimum combination scheme of water-fertilizer under condition of level-border and plastic film hole irrigation for corn. Appl. Mech. Mater. (2013) 405–408:2231–2237. 10.4028/www.scientific.net/AMM.405-408.2231

[B31] Sistani K. R. Baptiste M. J. Simmons J. R. (2014). Corn response to enhanced-efficiency nitrogen fertilizers and poultry litter. Agron. J. 106, 761–770. 10.2134/agronj2013.0087

[B32] Stanger T. F. Lauer J. G. (2008). Corn grain yield response to crop rotation and nitrogen over 35 years. Agron. J. 100, 643–650. 10.2134/agronj2007.0280

[B33] Sundaram P. K. Mani I. Lande S. D. Parray R. A. (2019). Evaluation of urea ammonium nitrate application on the performance of wheat. Int. J. Curr. Microbiol. Appl. Sci. 8, 1956–1963. 10.20546/ijcmas.2019.801.205

[B34] Sylvester-Bradley R. Kindred D. R. (2009). Analysing nitrogen responses of cereals to prioritize routes to the improvement of nitrogen use efficiency. J. Exp. Bot. 60, 1939–1951 10.1093/jxb/erp116 19395389

[B35] Venterea R. T. Bijesh M. Dolan M. S. (2011). Fertilizer source and tillage effects on yield-scaled nitrous oxide emissions in a corn cropping system. J. Environ. Qual. 40, 1521–1531. 10.2134/jeq2011.0039 21869514

[B36] Wang Y. Xu Z. Li B. N. Gao Q. Feng G. Z. Li C. L.. (2018). Effects of urea ammonium nitrate solution on grain yield and nitrogen uptake of spring maize in black soil region. Sci. Agric. Sin. 51, 718–727. 10.3864/j.issn.0578-1752.2018.04.011

[B37] Zhang A. Cui L. Pan L.i L. Hussain Q. Zhang X. Zheng J.. (2010). Effect of biochar amendment on yield and methane and nitrous oxide emissions from a rice paddy from Tai Lake plain, china. Agric. Ecosyst. Environ. 139, 469–475. 10.1016/j.agee.2010.09.003

[B38] Zhang F. S. Wang J. Q. Zhang W. F. Cui Z. L. Ma W. Q. Chen X. P.. (2008). Nutrient use efficiencies of major cereal crops in china and measures for improvement. Acta Pedol. Sin. 5, 915–924. 10.3321/j.issn:0564-3929.2008.05.018

[B39] Zhang W. F. Ma L. Huang G. Q. Wu L. Chen X. P. Zhang F. S. (2013). The development and contribution of nitrogenous fertilizer in China and challenges faced by the country. Sci. Agric. Sin. 46, 3161–3171. 10.3864/j.issn.0578-1752.2013.15.010

[B40] Zhang Y. H. Yao J. Bao D. J. He A. L. Luo X. S. Du J.. (2018). Effects of ammonium nitrate solution on yield, quality and nutrient uptake in maize. J. Agric. Sci. Technol. 20, 113–121. 10.13304/j.nykjdb.2017.0563

[B41] Zhang Y. H. Yao J. He A. L. Du J. Zheng C. F. Zhang J. M. (2017). Effects of the reducing and efficiency-increasing application of urea ammonium nitrate solution on the yield and nitrogen uptake and utilization of wheat. J. Henan Agric. Sci. 46, 6–12. 10.15933/j.cnki.1004-3268.2017.11.002

[B42] Zhang Z. S. Guo L. J. Liu T. Q. Li C. F. Cao C. G. (2015). Effects of tillage practices and straw returning methods on greenhouse gas emissions and net ecosystem economic budget in rice-wheat cropping systems in central China. Atmos. Environ. 122, 636–644. 10.1016/j.atmosenv.2015.09.065

[B43] Zhao B. Dong S. T. Zhang J. W. Liu P. (2010). Effects of controlled-release fertilizer on yield and nitrogen accumulation and distribution in summer maize. Acta Agron. Sin. 36, 1760–1768. 10.3724/SP.J.1006.2010.01760

[B44] Zhu J. X. He N. N. Wang Q. F. Yuan G. F. Wen D. Yu G. R.. (2015). The composition, spatial patterns, and influencing factors of atmospheric wet nitrogen deposition in Chinese terrestrial ecosystems. Sci. Total Environ. 511, 777–785. 10.1016/j.scitotenv.2014.12.038 25617702

